# Assessing fecal metaproteomics workflow and small protein recovery using DDA and DIA PASEF mass spectrometry

**DOI:** 10.20517/mrr.2024.21

**Published:** 2024-07-03

**Authors:** Angela Wang, Emily E F Fekete, Marybeth Creskey, Kai Cheng, Zhibin Ning, Annabelle Pfeifle, Xuguang Li, Daniel Figeys, Xu Zhang

**Affiliations:** ^1^Regulatory Research Division, Biologic and Radiopharmaceutical Drugs Directorate, Health Products and Food Branch, Health Canada, Ottawa K1A 0K9, Ontario, Canada.; ^2^Department of Biochemistry, Microbiology and Immunology, Faculty of Medicine, University of Ottawa, Ottawa K1H 8M5, Ontario, Canada.; ^3^School of Pharmaceutical Sciences, Faculty of Medicine, University of Ottawa, Ottawa K1H 8M5, Ontario, Canada.; ^#^Authors contributed equally.

**Keywords:** Fecal metaproteomics, microbiome, mass spectrometry, differential centrifugation

## Abstract

**Aim:** This study aims to evaluate the impact of experimental workflow on fecal metaproteomic observations, including the recovery of small and antimicrobial proteins often overlooked in metaproteomic studies. The overarching goal is to provide guidance for optimized metaproteomic experimental design, considering the emerging significance of the gut microbiome in human health, disease, and therapeutic interventions.

**Methods:** Mouse feces were utilized as the experimental model. Fecal sample pre-processing methods (differential centrifugation and non-differential centrifugation), protein digestion techniques (in-solution and filter-aided), data acquisition modes (data-dependent and data-independent, or DDA and DIA) when combined with parallel accumulation-serial fragmentation (PASEF), and different bioinformatic workflows were assessed.

**Results:** We showed that, in DIA-PASEF metaproteomics, the library-free search using protein sequence database generated from DDA-PASEF data achieved better identifications than using the generated spectral library. Compared to DDA, DIA-PASEF identified more microbial peptides, quantified more proteins with fewer missing values, and recovered more small antimicrobial proteins. We did not observe any obvious impacts of protein digestion methods on both taxonomic and functional profiles. However, differential centrifugation decreased the recovery of small and antimicrobial proteins, biased the taxonomic observation with a marked overestimation of *Muribaculum* species, and altered the measured functional compositions of metaproteome.

**Conclusion:** This study underscores the critical impact of experimental choices on metaproteomic outcomes and sheds light on the potential biases introduced at different stages of the workflow. The comprehensive methodological comparisons serve as a valuable guide for researchers aiming to enhance the accuracy and completeness of metaproteomic analyses.

## INTRODUCTION

The human gut microbiome contains an estimated 100 trillion microorganisms, including bacteria, fungi, protozoa, and viruses, which interact with each other and their host to foster a complex and dynamic environment^[1-3]^. The symbiotic host-microbial relationship of the gut microbiome is crucial to human health and contributes to many biological processes, such as metabolism, immunomodulation, *etc.*^[4]^. Many studies also suggest that microbiome dysbiosis is correlated with and may lead to the development of neurodegenerative, cardiovascular, metabolic, and gastrointestinal diseases, among others^[3,5-7]^. With the emerging importance of the gut microbiome in human health, disease, and therapeutics, studies on the microbiome, its taxa, and its products have become increasingly significant^[8]^.

Given the extremely high complexity of the microbiome, meta-omics approaches including metagenomics, metatranscriptomics, metabolomics, and metaproteomics, are commonly used in studying the microbiome composition and functions^[9,10]^. Among the different omics approaches, metaproteomics uses a mass spectrometer to directly measure the protein expressions and post-translational modifications (PTMs) of the microbial community^[11]^. Mass spectrometry (MS) analysis can be conducted with a data-dependent acquisition (DDA) or data-independent acquisition (DIA) strategy. DDA-based metaproteomics is commonly used due to its easy setup and analysis, flexibility, breadth of detection, and ability to relatively quantify chemically labeled peptides^[12]^. In a DDA mode, the most abundant ions from MS1 scan will be selected and fragmented during tandem MS scans; however, this data acquisition mode can risk losing information on the other less abundant peptides, particularly in complex samples such as microbiomes, which limits the depth, sensitivity, and reproducibility of metaproteomic data^[13,14]^. Contrastingly, DIA can sample all the peptides within the selected mass range and, therefore, theoretically can detect lower-abundance peptides in complex samples^[15]^. In the past few years, the application of DIA-MS-based proteomics was profoundly expanded due to these advantages, the advancement of bioinformatics tools, such as DIA-NN^[16]^, and advanced instrumental developments, such as timsTOF Pro^[17]^ and Astral MS analyzer^[18]^. More recently, the application of DIA-MS in metaproteomics has been reported, demonstrating great potential in increasing the depth of identification and accuracy of quantifications^[19-21]^.

One advantage of metaproteomics is the capability to measure non-bacterial components, including proteins originating from the host, as well as viral, fungal and archaeal species, without the need for additional experimental efforts^[22]^. This is particularly ideal for trans-kingdom, host-microbiome interaction studies^[23,24]^. The intestinal lumen is the home of diverse biotic and abiotic components, including host-secreted proteins such as antimicrobial proteins and proteins produced by microbes themselves, such as small microbial proteins. These small proteins/polypeptides, including a high proportion of antimicrobial peptides, are implicated in cell-signaling, transport, enzymatic activities, antitoxin systems, pathogenic colonization resistance, *etc.*^[25-27]^. Theoretically, these small proteins present in the intestinal samples can be identified in metaproteomics data; however, in practice, their identification is often overlooked due to suboptimal sample preparation and bioinformatics annotation steps, resulting in the loss of these portions of protein components^[28,29]^. Sample preparation methods, such as differential centrifugation, are commonly used to enrich and purify microbial cells from fecal sample debris and remove chemical contaminants that can impact downstream protein extraction and protein digestion^[30]^. However, the secreted small antimicrobial peptides from either the host or microbes may be lost during differential centrifugation.

It is well recognized that different sample processing methods such as the use of differential centrifugation, protein extraction methods, and protein digestion methods, and differences in data analysis workflows can yield different results and metaproteomic insights^[30-32]^. We have previously reported that physical disruption using bead beating or ultrasonication is needed to optimally extract proteins from Bacillota (previously Firmicutes)^[32]^. Tanca *et al.* showed that stool pretreatment by differential centrifugation significantly impacted metaproteomic observations^[30]^. However, there is still a need for further evaluation of the bias introduced by different steps of the experimental workflow as well as the emerging DIA data acquisition mode, and how they can contribute to the recovery of otherwise overlooked components, such as small antimicrobial proteins. Therefore, in this study, we conducted a comprehensive comparison of fecal sample preparation, protein digestion, data acquisition mode, and bioinformatic workflow using mouse feces to serve as a guide for metaproteomic experimental design.

## METHODS

### Mouse feces collection and differential centrifugation

Mouse fecal samples were collected from nine female C3H/HeN mice purchased from Charles River Laboratories, Senneville, Quebec, Canada. The animal procedures were approved by the Animal Care Committee in Health Canada and performed in accordance with institutional guidelines. Fecal samples were combined and crushed into a homogenous powder. This stock fecal sample was used for both differential centrifugation (DC) and non-differential centrifugation (NC) workflows.

Differential centrifugation of feces was performed according to a previous study^[32]^. Briefly, 0.5 mL glass beads (BioSpec, Cat#11079125) and 7.5 mL cold phosphate-buffered saline (PBS) per gram of fecal sample were added to the samples, followed by vortexing and centrifugation at 300 g at 4 °C for 5 min to collect the supernatant. The remaining fecal pellets were extracted three more times with 7.5, 5, and 5 mL cold PBS, and the fecal pellet at the end of the 4 extractions was discarded. Once pooled, additional debris from the extractions was removed by three centrifugations at 300 g at 4 °C for 5 min. The supernatant extract was then spun down at 14,000 g at 4 °C for 20 min to collect the microbial pellet. The microbial pellet was washed twice with cold PBS by resuspending and centrifuging at 14,000 g at 4 °C for 20 min, then frozen until use.

### Protein extraction, trypsin digestion and desalting

#### Sample lysis

NC feces were lysed by directly resuspending dry, frozen, crushed fecal pellet in lysis buffer at a ratio of 20 mg dry fecal powder material / 1 mL lysis buffer. DC microbial pellets were lysed by resuspending the wet microbial pellets with a ratio of 100 mg wet pellets / 1 mL lysis buffer (assuming that 20% of the wet pellets are dry materials). The same lysis buffer was used for all samples, consisting of 4% (w/v) sodium dodecyl sulfate (SDS), 8 M urea, and 1× Halt protease inhibitor single-use cocktail (Thermo Scientific, Cat#78425) in 50 mM Tris-HCl (pH 8). Sample lysates were sonicated using a QSonica Q700 water-chilled cup-horn sonicator at 50% amplitude, 10 s pulse on/off cycle, for 20 min active sonication time at 8 °C. The lysate was then centrifuged at 16,000 g for 10 min at 8 °C to remove any non-lysed debris. Protein-containing supernatant was transferred to a new tube and protein concentrations were determined using the Pierce BCA protein assay kit (Thermo Fisher Scientific, Cat#23225) following the manufacturer’s protocol.

#### In-solution trypsin digestion

For in-solution trypsin digestion, proteins underwent acetone precipitation by adding 5 volumes of protein precipitation buffer composed of 50% acetone, 50% acetonitrile (ACN), 0.1% acetic acid, mixed through inversion and incubated at -20 °C overnight. Samples were spun down at 16,000 g at 4 °C for 25 min and the supernatant was discarded. The remaining pellet was washed with ice-cold acetone, sonicated with a QSonica Q700 water-chilled cup-horn sonicator at 50% amplitude for 10 s, and spun down at 16,000 g at 4 °C for 10 min for a total of 3 washes. After briefly air drying, the protein pellets were resuspended in 6 M urea in 50 mM ammonium bicarbonate (ABC) buffer (pH 8) for trypsin digestion. Protein concentrations were determined using the Pierce BCA protein assay kit, and 100 µg of protein lysates of each sample were reduced with 10 mM 1,4-dithiothreitol (DTT) incubated at 850 rpm at 56 °C for 30 min and alkylated with 20 mM iodoacetamide (IAA) for 40 min at room temperature protected from light. Samples were diluted in 50 mM ABC buffer to a final urea concentration of 0.6 M and then digested with 4 µg MS-grade Trypsin (Promega, Cat#V511B, lot: 0000575718) / 100 µg of protein incubated at 850 rpm at 37 °C for 20 h. The reaction was stopped by adding formic acid (FA) to acidify the samples to pH 2-3 prior to desalting, as described below.

#### Filter-aided sample preparation digestion

For filter-assisted digestion methods, two different molecular weight cut-off filters were used, namely filter-aided sample preparation (FASP)-10kDa and FASP-3kDa (Merck Millipore Ltd). For each sample, 100 µg of protein lysate was directly diluted to 200 µL of 8 M urea in 100 mM Tris-HCl buffer and added to the pre-rinsed FASP columns. SDS was diluted twice using 200 µL of 8 M urea in 100 mM Tris-HCl buffer each time. Reduction was performed by adding 8 M urea in 100 mM Tris-HCl buffer containing a final concentration of 20 mM DTT and incubated at 850 rpm at 37 °C for 30 min. After removing the flow-through, samples were then alkylated by 20 mM IAA at room temperature in the dark for 30 min. To remove excessive IAA, an additional 200 µL 20 mM DTT in 8 M urea in 100 mM Tris-HCl buffer was added, incubated at room temperature for 2 min, and eluates were discarded. The column was then washed with 8 M urea in 100 mM Tris-HCl buffer once and 100 mM Tris-HCl buffer for four times prior to adding 200 µL 100 mM Tris-HCl buffer containing 4 µg of trypsin (1 µg trypsin : 25 µg protein input). The trypsin digestion was performed by shaking at 850 rpm at 37 °C for 20 h. Peptides were eluted by spinning at 16,000 g at room temperature for 20 min followed by additional elution with 200 µL fresh 100 mM Tris-HCl buffer. Both eluents were combined and acidified to a pH of 2-3 using 10% (v/v) FA for desalting.

#### Desalting

Desalting was performed using C18 columns (Thermo Scientific, Cat#89870). Columns were activated by adding 100% ACN, centrifuging at 100 g for 1 min for a total of 3 times, and then equilibrated with 0.1% (v/v) FA, centrifuging at 300 g for 2 min for 2 times. Samples were loaded to the column and centrifuged at 300 g to remove flow-through until all samples were loaded. The desalting column was washed with 0.1% (v/v) FA for 2 times. Desalted peptides were then eluted with 100 µL 80% (v/v) ACN / 0.1% (v/v) FA buffer for two times by centrifuging at 100 g for 1 min for each elution. The eluates containing desalted tryptic peptides were then dried on a centrivap (Labconco, Cat#7810010) and stored at -20 °C until LC-MSMS analysis.

### LC-MSMS analysis

Dried tryptic peptides were resuspended in 0.1% (v/v) FA to a final concentration of 1µg/µL, and 3 µg peptides were loaded for MS analysis using a timsTOF Pro 2 mass spectrometer (Bruker Daltonik, Bremen, Germany) coupled to a nanoElute 2 UPLC system (Bruker Daltonik). The instrument was calibrated prior to analysis with Chip Cube High Mass Reference Standard (Agilent, Cat#G1982-85001). A two-column system of HPLC was used consisting of a C8 trap column before separating on a PepSep Twenty-five analytical column (25 cm × 75 μm column packed with 1.9 μm C18 particles) (Bruker Daltonik, Cat#1893477). Chromatographic separation was achieved at a flow rate of 0.5 µL/min over 48 min in linear steps as follows (solvent A was 0.1% FA in water, solvent B was 0.1% FA in ACN): initial, 2%B; 40 min, 35%B; 40.5 min, 95%B; 45 min, 95%B; 48 min, 95%B. The eluting peptides were analyzed in either data-dependent acquisition coupled with parallel accumulation-serial fragmentation (DDA-PASEF) mode or data-independent acquisition coupled with PASEF (DIA-PASEF) mode in the timsTOF Pro 2 mass spectrometer.

For DDA-PASEF mode, a MS survey scan of 100-1,700 m/z and ion mobility range of 0.85-1.30 Vs/cm^2^ was performed in the timsTOF MS. During the MS/MS scan, 4 PASEF ramps were run with an intensity threshold of 2,500, target intensity of 20,000, and a maximum precursor charge of 5. The TIMS analyzer was operated in a 100% duty cycle with equal accumulation and ramp times of 100 ms each and a total cycle time of 0.53 s. The collision energy was ramped linearly as a function of mobility from 59 eV at 1/k0 = 1.6 Vs/cm^2^ to 20 eV at 1/k0 = 0.6 Vs/cm^2^.

For DIA-PASEF mode, a MS survey scan of 100-1,700 m/z with an ion mobility range of 0.6-1.60 Vs/cm^2^ was performed. The TIMS analyzer was operated in a 100% duty cycle with equal accumulation and ramp times of 100 ms each and a total cycle time estimated at 1.8 s. During DIA-PASEF MS/MS scan, precursors with m/z between 400 and 1,200 were defined in 16 scans containing 32 ion mobility steps with an isolation window of 26 Da in each step with 1 Da overlap with neighboring windows. The collision energy for DIA-PASEF scan was ramped linearly from 59 eV at 1/k0 = 1.3 V·s/cm^2^ to 20 eV at 1/k0 = 0.85 V·s/cm^2^.

### Bioinformatic data processing

#### Spectral library and reduced database generation

The mouse gut microbial gene catalog database, containing ~2.6 million nonredundant protein sequences, was downloaded from GigaScience Database (http://gigadb.org/dataset/100114)^[33]^. The reviewed mouse UniProtKB database was downloaded from UniProt (downloaded on 2024/01/01, 17,179 protein entries). These two databases were combined as the starting original database for generating a spectral library or a reduced FASTA database using the DDA-PASEF dataset, employing either pFind^[34]^ or MSFragger^[35]^. For pFind search, MS raw files were first converted from .d to .mgf files using MSconvert (v3.0.23240). The resulting MGF files were then searched using open search mode against the combined database with the following parameters: enzyme: Trypsin KR_C, number of missed cleavages: 3, precursor and fragment tolerance: +/- 20 ppm, and an false discovery rate (FDR) of < 1% at both peptide and protein levels. The protein sequences of all identified proteins in the pFind search, including indistinguishable proteins, were extracted from the original database to generate a pFind-generated reduced database using an in-house Perl script. For MSfragger search, FragPipe (v20.0) was used with either the full combined database or the pFind-generated reduced database for generating spectral libraries that will be used for analysis for the DIA-PASEF dataset. For MSfragger search using the full combined database, a database split factor of 10 was used. Both searches followed the default workflow and used ciRT for spectral library generation with a peptide and protein level FDR threshold of 1%. In addition to spectral libraries generated, the protein sequences of all identified proteins, including indistinguishable proteins, of MSFragger search against the original database were extracted using an in-house Perl script to generate a MSFragger-derived reduced FASTA database.

#### DIA-NN search for identification and quantitation of DIA-PASEF data

DIA-PASEF data were processed with DIA-NN^[16]^ (v1.8.1) using either spectral libraries generated through MSFragger or a library-free mode using reduced FASTA databases generated through pFind or MSFragger searches as described above, with a precursor FDR threshold of 1%. Default settings were used for all the DIA-NN searches.

#### MSFragger search for identification and quantitation of DDA-PASEF data

To obtain identification and quantitation results of the DDA-PASEF dataset, the DDA data were processed with the FragPipe (v21.1) MSFragger node using the LFQ-MBR workflow. The full mouse gut microbial gene catalog and host database were used. Similar to the spectral library generation workflow, a split database strategy was used to improve the sensitivity of peptide-spectrum match (PSM) with a database split factor of 10. MaxLFQ intensity of identified protein groups was used in this study with a minimum ion of 1.

#### Taxonomic and functional annotation and analysis

Taxonomic and functional annotation and analysis for both DIA-NN and MSFragger outputs were performed using MetaLab^[36]^ (version 2.3). Briefly, the DIA-NN outputs report_pg.tsv and report_pr.tsv files were used for functional and taxonomic annotations, respectively. Similarly, the MSFragger outputs combined_proteins.tsv and combined_peptides.tsv were used for functional and taxonomic annotations, respectively. Unipept option in MetaLab was used to generate taxonomic annotations for both database search results, and a minimum of three distinct peptides was used for confident identification of taxa.

#### Database search for small proteins and antimicrobial peptides

A small protein database derived from the human gut microbiome was downloaded from the supplemental data of a previous study by Sberro *et al.*^[25]^. We used the protein cluster data table, which contained 444,054 entries, to generate the small protein database. The AMPsphere AMP database was downloaded on September 1, 2024, from AMPsphere (https://ampsphere.big-data-biology.org/home), containing 863,498 entries^[37]^. To enable the calculation of the relative abundance of small protein or antimicrobial peptides (AMPs) to total proteins in a sample, all identified peptide sequences from previous search using the combined database (gut microbial gene catalog and mouse proteome) were concatenated with the AMP or small protein databases for DIA-NN search or MSFragger search for DIA-PASEF data or DDA-PASEF data, respectively. DDA and DIA data were quantitatively analyzed using the methods described above with the respective alternate databases.

### Data visualization and statistical analysis

Experimental flowcharts were generated using BioRender (https://www.biorender.com/). Graphs were generated using R package ggplot2 and ggpubr.

## RESULTS

### Evaluating bioinformatics workflows for DIA-PASEF metaproteomics

**Figure 1:**
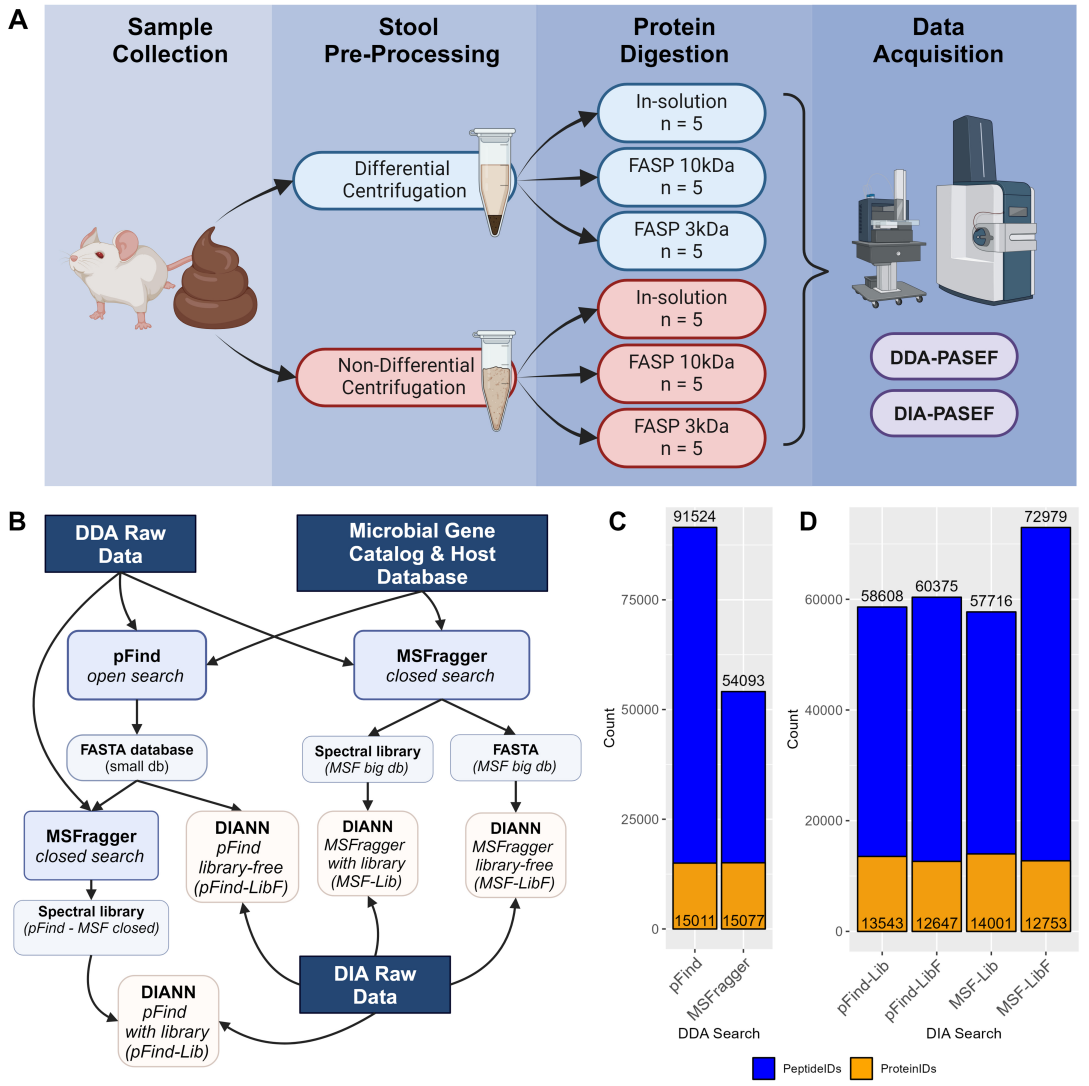
Experimental Overview and evaluation of bioinformatics workflow. (A) Flowchart depicting mouse fecal sample processing workflow and MS data acquisition. (B) Flowchart depicting different bioinformatic workflows tested in this study. Histograms of the total number of peptides and proteins identified with the different bioinformatics processing workflows as shown in (B) for (C) DDA and (D) DIA datasets, respectively. MS: Mass spectrometry; DDA: data-dependent acquisition; DIA: data-independent acquisition.

To evaluate fecal sample preparation workflows, a pooled fecal sample from C3H/HeN female mice was crushed into a powder, homogenized and aliquoted for either microbial enriched protein extraction through DC or direct protein extraction with NC workflow [Figure 1A]. To assess the potential impacts of protein digestion methods, both DC and NC protein lysates were subjected to in-solution trypsin digestion (following acetone precipitation for detergent removal), FASP with 10kDa molecular weight cut-off filter (FASP10), or 3kDa filter (FASP3). All comparisons were conducted with five replicates with a total of 30 peptide samples for MS analysis on a timsTOF Pro 2 mass spectrometry system using both DDA- and DIA-PASEF acquisition modes [Figure 1A].

A major challenge for DIA metaproteomic data analysis is that bioinformatics tools, such as DIA-NN, cannot handle large databases. Currently, most workflows utilize DDA data of the same or representative samples to generate a spectral library from the original protein databases. We therefore first evaluated the use of two widely used database search engines for DDA data, pFind (open search) and MSFragger (closed search with split databases), to generate reduced databases from the mouse gut microbial gene catalog database [Figure 1B]. The pFind open search for all 30 DDA data files identified 91,524 peptides corresponding to 15,011 protein groups in total, while the MSFragger search for the 30 DDA data files identified 54,093 peptides corresponding to 15,077 protein groups [Figure 1C]. DIA-NN searches with spectral library or library-free modes from either pFind or MSFragger reduced databases were then conducted to see which pipeline for DIA data processing yields the best identification results. Briefly, protein sequences were extracted from both search outputs to generate reduced FASTA databases for DIA-NN search with library-free mode (pFind-LibF for using pFind-generated protein database, and MSF-LibF for using MSFragger-generated database). Spectral libraries were generated by MSFragger with either the full database or a pFind-generated reduced protein database, and were used for DIA data analysis (MSF-Lib and pFind-Lib, respectively). As shown in Figure 1D, all DIA-NN search inputs yielded similar protein group numbers, falling between 12,647 and 14,001. The main difference in identification was found in the peptide level, where all workflows tested resulted in approximately 58,000-60,000 peptides except for MSF-LibF which identified 72,979 peptides in total for the DIA-PASEF dataset. Based on these evaluations, we then chose MSF-LibF workflow for DIA-PASEF data processing, and to be consistent, the MSFragger LFQ-MBR quantification using the full database (with 10 splits) was used for the analysis of the DDA-PASEF dataset.

### DIA-PASEF metaproteomics achieved better identification and quantification

**Figure 2:**
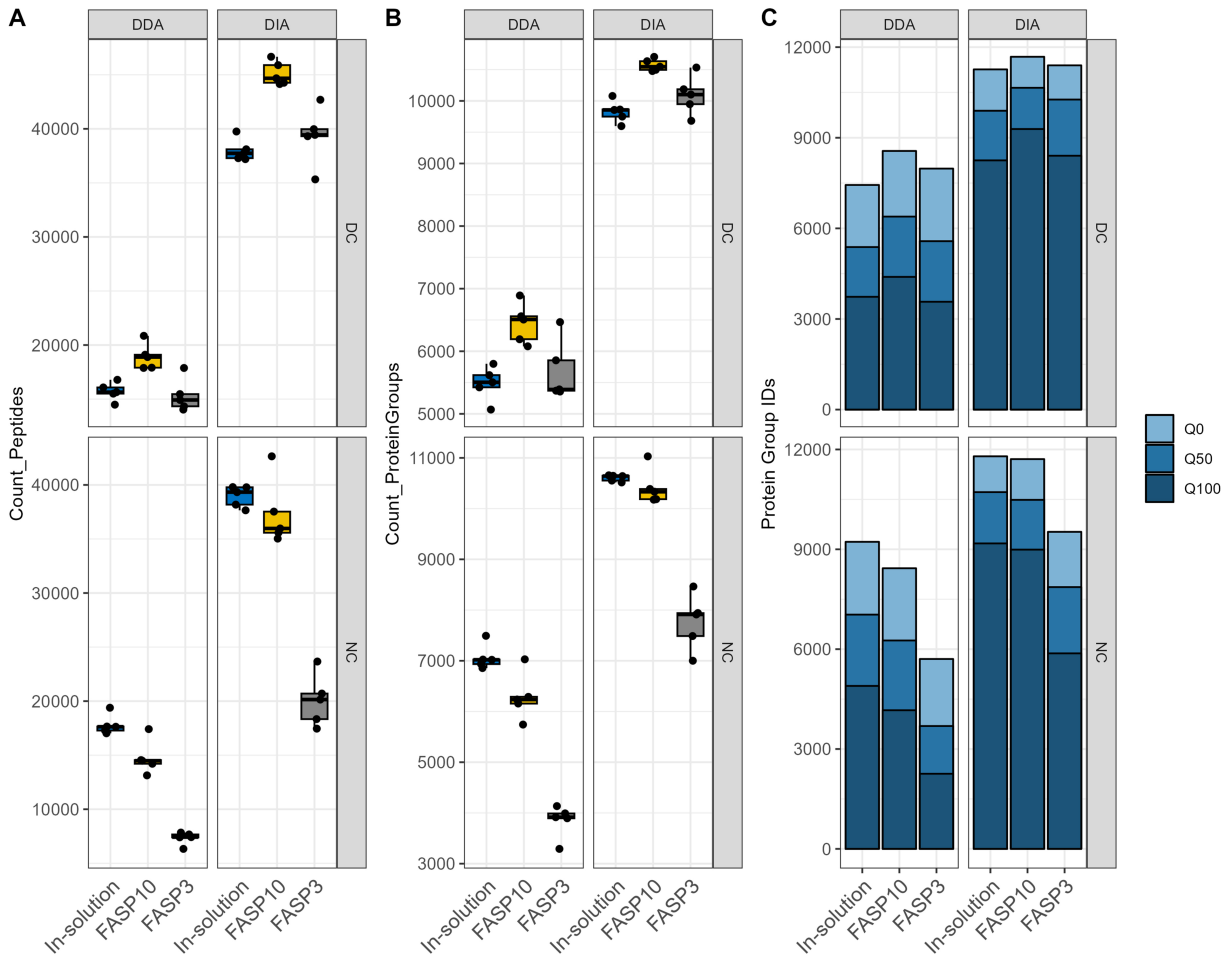
Comparison of identification and quantification abilities of DDA and DIA-PASEF approaches. Number of peptides identified by DDA and DIA-PASEF (A) mass spectrometry methods. Number of protein groups identified by DDA and DIA-PASEF (B) mass spectrometry methods. Number of quantitated protein groups in any, half, or all (Q0, Q50, and Q100, respectively) samples in each group identified by DDA- or DIA-PASEF (C) mass spectrometry. For each figure panel, facet_grid function in ggplot2 was used to generate different sections; DC groups are at the top and NC at the bottom, while DDA groups are on the left side and DIA on the right side. DDA: Data-dependent acquisition; DIA: data-independent acquisition; PASEF: parallel accumulation-serial fragmentation; DC: differential centrifugation; NC: non-differential centrifugation.

We first compared the DDA-PASEF and DIA-PASEF data acquisition modes in terms of peptide and protein identification, as well as protein quantification in fecal metaproteomics. Across all fecal sample preparation methods, there was a consistent increase in peptides and protein groups identified per sample when samples were run using DIA-PASEF mass spectrometry compared to those run with DDA-PASEF mode [Figure 2A and B]. This finding is in agreement with a previous study by Gómez-Varela *et al.*^[21]^. For the identification of peptides and protein groups, the digestion method using a FASP-10kDa approach had a slight edge over other digestion methods tested in DC preparations, and in-solution digestion had a slight edge in NC samples [Figure 2A and B]. DIA-PASEF runs for DC samples achieved the highest number of precursor identifications with nearly 50,000 precursors per sample (identifications ranging from 36,912 to 49,928 precursors, 35,324 to 46,665 peptides, 9,598 to 10,705 protein groups), while nearly 21,000 peptides (identifications ranging from 14,029 to 20,852 peptides, to 5,068 to 6,891 protein groups) were identified for the same samples with DDA-PASEF runs. DIA-PASEF runs for NC samples achieved a competitive number of precursor identifications with nearly 45,000 precursors per sample (identifications ranging from 17,681 to 44,915 precursors, 17,457 to 42,646 peptides, 7,002 to 11,031 protein groups), while up to almost 20,000 peptides (identifications ranging from 6,334 to 19,389 peptides, 3,292 to 7,492 protein groups) were identified for the same samples with DDA-PASEF runs. The filter-assisted sample preparation, using the FASP-3kDa columns, showed comparable identification rates for DC samples compared to other digestion methods, but it led to a decrease in peptide and protein group identifications when applied to NC samples [Figure 2A and B]. The identified peptides in the NC with the FASP-3kDa group exhibited smaller average peptide lengths and fewer missed cleavage sites compared to the other groups [Supplementary Figure 1]. These differences might be due to the potential undesirable interactions between the FASP column matrix and non-protein components in NC samples and the resulting low efficiency for eluting large peptides.

The impacts of MS data acquisition and sample preparation on quantification were then assessed with the amount of missing values across samples in each group. In Figure 2C, Q0 indicates a protein group with quantified intensity in at least 1/5 replicates in the group, Q50 is present in at least 3/5 replicates, and Q100 is present in all replicates. Along with an increased number of protein group identifications in DIA-PASEF data across all sample preparation methods, there is a distinctly higher proportion of protein groups quantified in all sample replicates (Q100) for DIA-PASEF data compared with DDA-PASEF, in particular for samples prepared with in-solution digestion and FASP-10K column (73%-80% in DIA *vs.* 49%-53% in DDA; Figure 2C). Evaluation of the intra-group sample-wise Pearson’s correlation of the quantified protein intensities indicates high quantitative reproducibility in both DDA and DIA datasets for all proteins (Pearson’s r of 0.89-0.96 and 0.90-0.97, respectively) and for low abundant small proteins (100 amino acid length threshold; Pearson’s r of 0.82-0.96, 0.84-0.97, respectively) [Supplementary Figure 2]. Therefore, DIA-PASEF mass spectrometry methods for mouse metaproteomic samples from various preparations provide more identifications with fewer missing values, enhancing the potential for quantitative analysis.

### DIA-PASEF for non-enriched samples yielded the highest recovery of small proteins and antimicrobial peptides

When conducting the database search, we included both microbiome and host protein sequences. When looking at host proteins, DIA-PASEF mass spectrometry methods identified a higher number of host proteins per sample than DDA; this outcome was anticipated, as DIA-PASEF identified more proteins. Despite an increased diversity of host proteins identified in the DIA-PASEF dataset for each sample, the relative abundance of host proteins per sample did not show an obvious difference between both data acquisition and protein digestion methods [Supplementary Figure 3]. Differential centrifugation does not appear to have a major impact on the identification count of host proteins or their relative abundances, which is different from a previous study with human feces^[30]^. This might be due to the relatively low amount of host protein (less than 18% in abundance) in the feces of healthy mice that were used in this study [Supplementary Figure 3]. The most abundant host proteins identified in the fecal samples include those involved in digestion function and antimicrobial activity, such as regenerating islet-derived protein 3-beta (Reg3β) and immunoglobins, which may bind tightly to the microbe surface and thereby become enriched with microbial cells.

**Figure 3:**
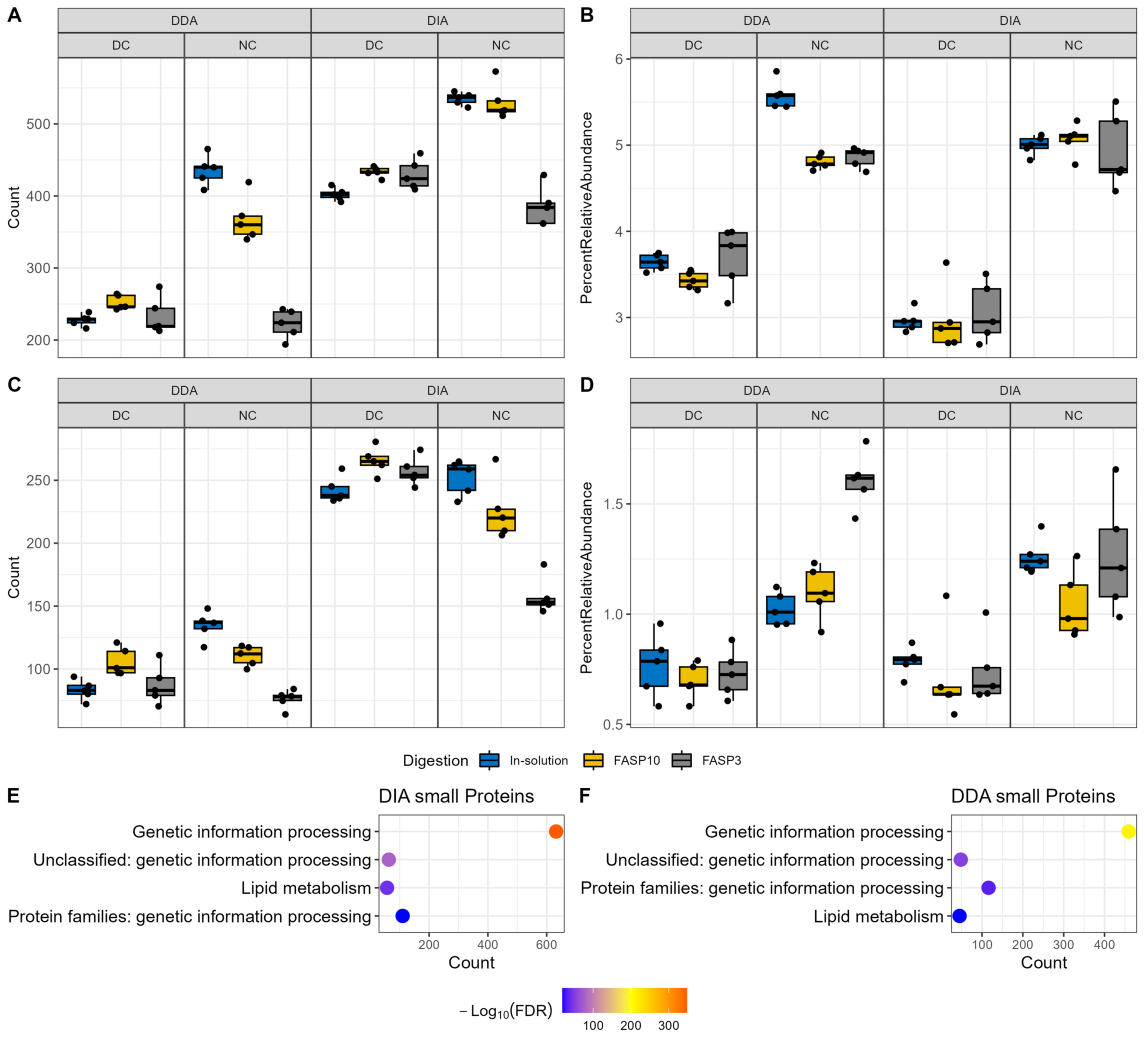
Quantitation of small proteins and antimicrobial peptides in mouse fecal samples. (A) Count and (B) relative abundance of small proteins in samples using a 100 amino acid cut-off. Functional enrichment analysis of identified small proteins in (C) the DDA-PASEF dataset and (D) the DIA-PASEF dataset, using all identified proteins as background in DDA and DIA datasets, respectively. Functional annotation was performed using GhostKOALA, and only significantly enriched functions were shown (adjusted *P* value ≤ 0.05). (E) Count and (F) relative abundance of AMPs when searched against the AMPsphere database. Facet_grid function in ggplot2 was used to generate different sections; DC and DDA groups are on the left side, while NC and DIA on the right side. DDA: Data-dependent acquisition; PASEF: parallel accumulation-serial fragmentation; DIA: data-independent acquisition; AMPs: antimicrobial peptides; DC: differential centrifugation.

Next, we evaluated whether different sample preparation methods and MS data acquisition modes could impact the identification of small proteins that are commonly overlooked in classical metaproteomics. Among all the identified proteins in this dataset, there were 31 protein groups with ≤ 50 amino acids and 845 with ≤ 100 amino acids. With the application of recent more sensitive mass spectrometers, > 2,000 small proteins (< 100 amino acids) can be identified in a similar experimental system^[38]^. We selected a 100 amino acids cut-off in this study to facilitate comparison between groups^[28,39]^. There were 200-600 small proteins identified per sample, with DIA-PASEF yielding higher identifications than DDA-PASEF mode [Figure 3A]. Differential centrifugation reduced the number of small protein identifications when the extracted proteins were digested using in-solution and FASP-10kDa methods, but not for those with FASP-3kDa method, which may be due to the lower overall performance of FASP-3kDa for NC samples. When considering the sum relative abundance of those small proteins, the type of digestion, FASP column size and MS acquisition mode all present minimal impacts [Figure 3B]. We then performed functional and taxonomic annotation using GhostKOALA for these identified small proteins, which showed that they are significantly enriched in genetic information processing and lipid metabolism pathways [Figure 3C and D, Supplementary Figure 4]. In addition, the identified small proteins in both DDA and DIA datasets were significantly enriched in undefined taxa (adjust *P* value of 1.59E-68 and 1.08E-45 for DDA and DIA datasets, respectively) from the microbiomes [Supplementary Figure 5]. This might be due to the fact that small protein sequences have lower information content for taxonomic annotation and are being under-represented in current knowledge databases. Altogether, this study showed that the use of differential centrifugation depleted the abundance of small proteins within sample sets across both DDA and DIA acquisition modes and protein digestion methods, suggesting that the NC method is superior when targeting and investigating small proteins specifically. It is worth mentioning that the NC method also has the advantage of shortened sample preparation steps and time, and thereby reduces the sample-to-sample variations introduced during sample preparation.

Previous metagenomics data mining identified small open reading frames (sORF) encoding > 4,000 small proteins (≤ 50 amino acids) in the human microbiome, with the majority having no known function^[25]^. Although the human microbiome may not fully encapsulate the small proteins that would be present in a mouse microbiome, the overlap that exists can help reinforce the evaluation of the impacts of different sample preparation methods and MS data acquisition mode on small protein identification. To enable calculation of the relative abundance and mitigate false discovery, all identified mouse gut microbial peptide sequences in this study were combined with the predicted small protein sequences for database search for both DDA and DIA-PASEF data using MSFragger and DIAN-NN, respectively. A consistent and large increase in small protein identifications in all sample preparation methods was observed when samples were run using a DIA-PASEF mode than with DDA-PASEF regardless of upstream sample preparation workflows [Supplementary Figure 6]. NC combined with FASP-3kDa digestion workflow tends to identify the lowest number of small proteins, which may be due to the overall low protein identifications in this group; however, the small proteins identified represented the largest percentage in abundance in samples. In fact, for both in-solution and FASP digestion workflows, NC leads to an increased abundance of small proteins regardless of protein digestion methods and MS acquisition modes, suggesting that differential centrifugation depletes small proteins within the sample. Altogether, the findings in this study suggest that direct fecal lysis for protein extraction, followed by digestion with either in-solution or FASP-10kDa workflows, and quantification using DIA-PASEF presented the highest number of identifications and relative abundance of small proteins within the sample [Supplementary Figure 6].

Small proteins are implicated in diverse functions of the microbiome, including defense against other microbes or pathogens^[26]^. Among the predicted small proteins in the human microbiome, around 30% were predicted to be secreted or transmembrane proteins and 39 protein families were predicted to be novel AMPs^[25]^. By using a deep learning approach, Ma *et al.* identified 2,349 potential AMPs from human microbiome metagenomic data and preliminary biological validation showed > 80% positive rate^[27]^. More recently, Santos-Júnior *et al.* leveraged a vast dataset of more than 1.5 million metagenomes or microbial genomes of diverse origins to establish a prokaryotic AMP database, AMPSphere^[37]^, which consists of 863,498 predicted AMPs. To evaluate the impacts of a metaproteomic workflow for AMP identification, we re-searched both DDA and DIA datasets using the AMPSphere database concatenated with all identified gut microbial peptide sequences. Consistent with total and small protein identification, DIA-PASEF showed a higher AMP identification (200~300 AMPs, excluding the FASP-3K group) compared to DDA-PASEF methods (50~150 AMPs) [Figure 3E and Supplementary Figure 7]. When looking at the relative abundance of AMPs within each sample, it appears that differential centrifugation decreases the abundance of AMPs [Figure 3F]. Evaluation of the sample-wise Pearson’s correlation of the quantified AMP intensities indicates higher correlations intra-groups compared to inter-groups in both DDA and DIA datasets [Supplementary Figure 2E and F]. The findings suggest that NC samples with digestion methods of either in-solution and FASP-10kDa followed by MS analysis with DIA-PASEF mode resulted in high AMP diversity observed at a high relative abundance.

### Over-representation of *Muribaculum* in mouse fecal metaproteome with differential centrifugation

**Figure 4:**
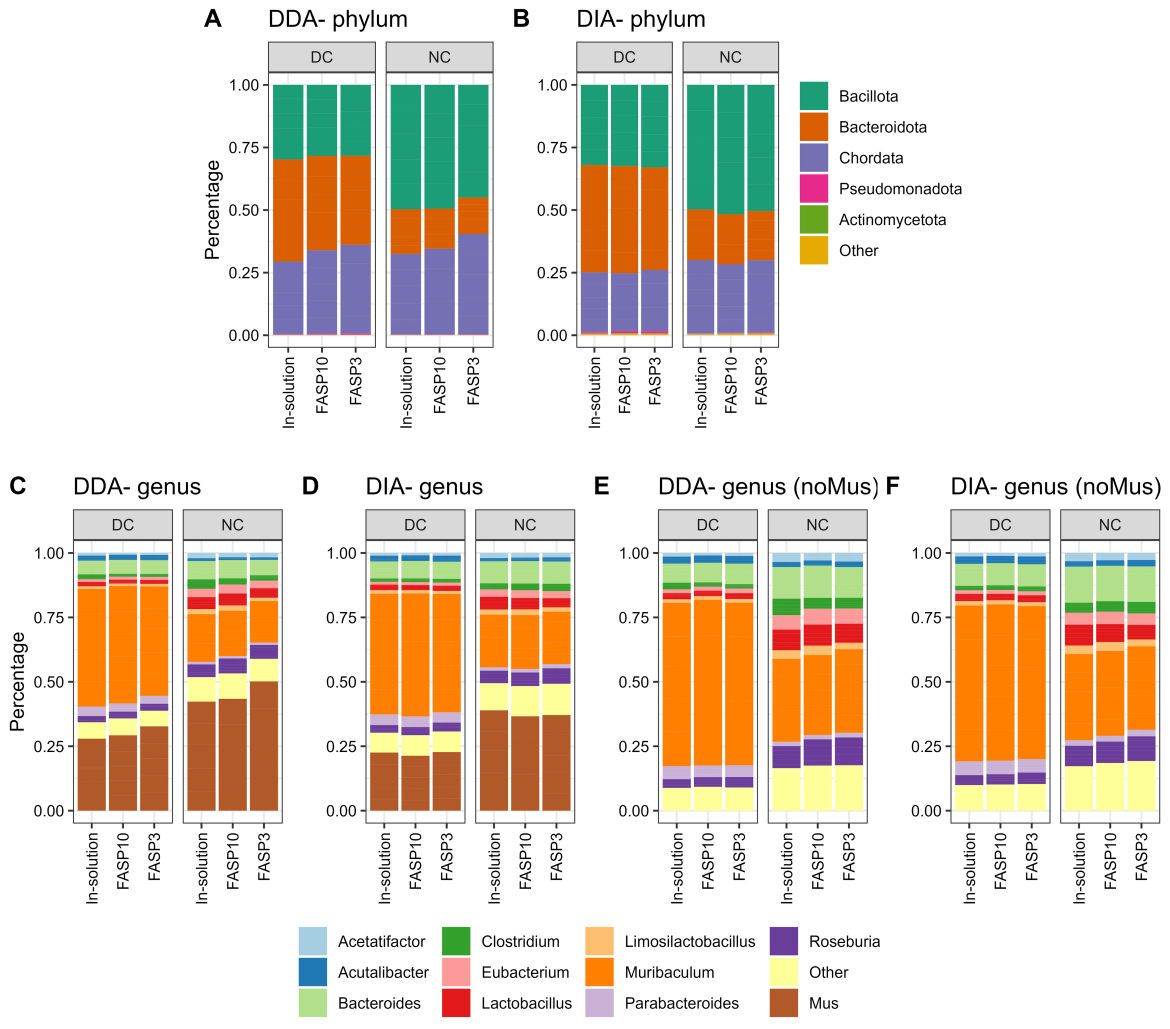
Taxonomic profiles of mouse fecal metaproteome. (A and B) Phylum level, (C and D) genus level with host (*Mus*), or (E and F) without host were shown. The group mean relative abundance of phyla or genera was used for plotting. Top 5 phyla or top 10 genera were shown, with the remainder grouped as “Other” in the stacked plots. Facet_grid function in ggplot2 was used to generate different sections; DC groups are on the left side and NC on the right side. DC: Differential centrifugation; NC: non-differential centrifugation.

We next evaluated whether different sample preparation methods and MS data acquisition mode impact the taxonomic profiles using metaproteomics. By using a threshold of a minimum of three distinctive peptides for confident taxon identification, this study identified 12 phyla, 13 classes, 21 orders, 23 families, 62 genera, and 96 species in the DDA-PASEF dataset, and 17 phyla, 22 classes, 19 orders, 32 families, 70 genera, and 96 species in the DIA-PASEF dataset. Here, we selected the phylum and genus levels for the evaluation. No obvious difference in the abundance distribution of abundant taxa was observed between DDA and DIA datasets at both phylum and genus levels [Figure 4]. The major differences were observed between DC and NC, which is in agreement with previous studies of both human and mouse microbiomes^[30,40]^. A higher relative abundance of *Chordata or Mus* (host) was observed in NC groups compared to DC groups, in particular at the genus level [Figure 4A-D]. Interestingly, we observed a difference in *Mus* (host) relative abundance between different protein digestion methods in DDA-PASEF data, but not in DIA-PASEF data [Figure 4C and D, Supplementary Figure 8]. On the contrary, the microbial compositions were highly consistent in both DDA-PASEF and DIA-PASEF datasets.

Metaproteomics analysis demonstrated that *Bacillota* (previous *Firmicutes*) and *Bacteroidota* (previous *Bacteroidetes*) were the two predominant phyla in mouse feces, but the relative abundances of these two phyla were dramatically altered by the use of differential centrifugation during sample pre-processing. Marked higher relative abundances of *Bacteroidota* and lower abundances of *Bacillota* were observed in DC samples compared to NC in both DDA and DIA datasets. This is in agreement with previous studies on mouse metaproteomes^[40]^. However, the direct opposite of changes was reported in human microbiomes where lower levels of *Bacteroidota* and higher levels of *Baccilota* were obtained when samples were prepared using differential centrifugation^[30]^. This difference might be due to the known marked different bacterial genus/species compositions of mice and human microbiomes. As shown in Figure 4E and F, *Muribaculum* was the genus mainly driving the elevation of *Bacteroidota* in DC samples, which represent ~70% of the microbial abundance in the samples. *Muribaculum* species are known to be dominant in mouse gut microbiota, but were only recently well characterized and demonstrated to have very high host preference (prevalence of 67% in mice compared to 7% in human gut)^[41]^. In contrast, in human gut microbiota, the genus *Bacteroides* is usually the most abundant in *Bacteroidota*^[42]^. In this study, we found that the relative abundance of *Bacteroides* was lower in DC compared to NC, which is in agreement with the observations in human gut microbiota. It is unknown why *Muribaculum* displayed different responses to DC compared to their neighboring genera within the sample phylum *Bacteroidota*, but this observation suggests that sample preparation methods need to be optimized for microbiomes of different origins and the microbial species of interest for a particular study.

### Differential centrifugation altered the functional profiles of fecal metaproteomes

**Figure 5:**
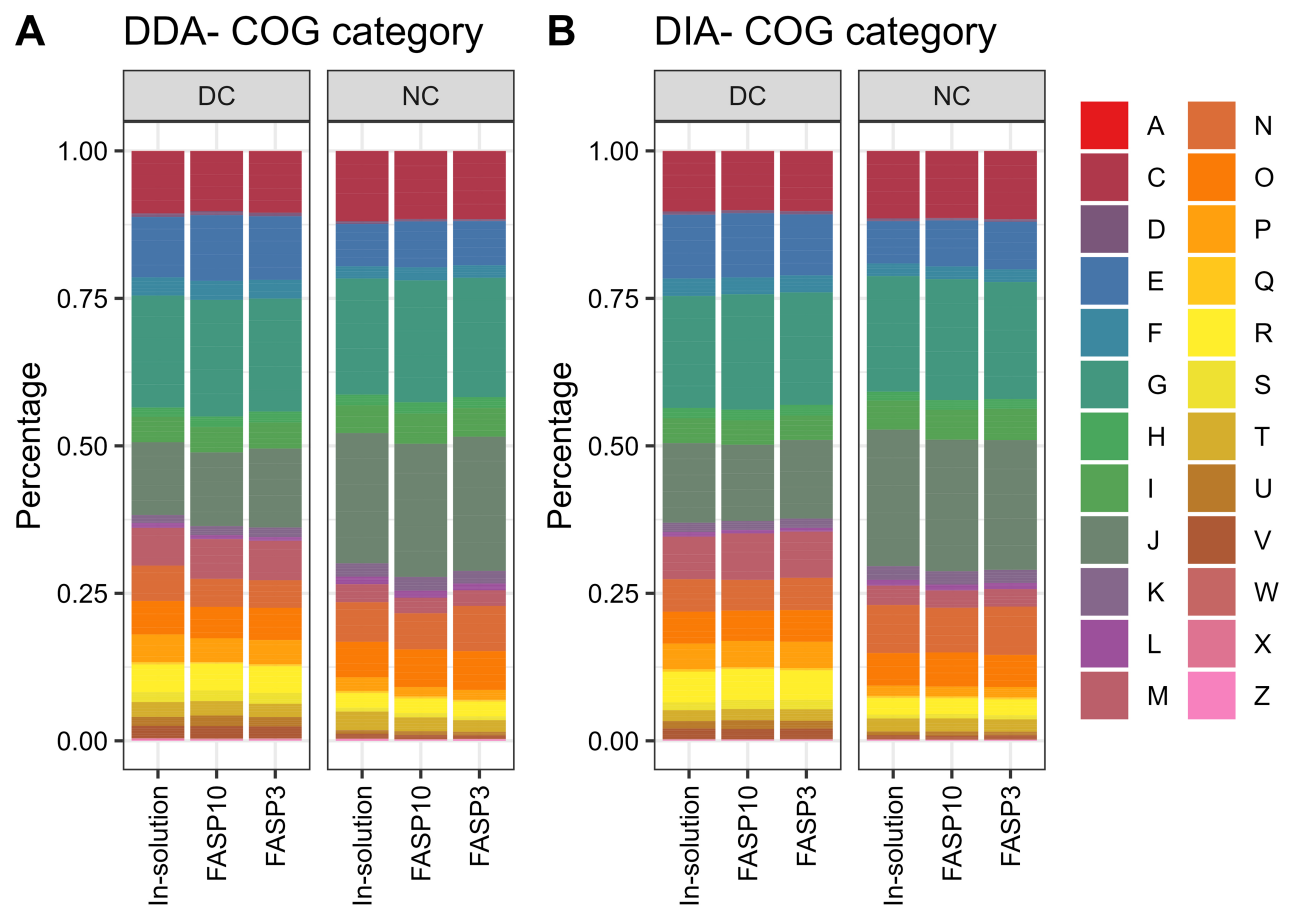
Composition of identified COG categories in mouse fecal metaproteome. The group mean relative abundance of COG category was plotted for (A) DDA and (B) DIA dataset, respectively. Each letter represents a COG category according to the Database of COGs https://www.ncbi.nlm.nih.gov/research/cog/. COG: Clusters of Orthologous Gene; DDA: data-dependent acquisition; DIA: data-independent acquisition.

Lastly, we evaluated the influence of sample preparation methods and MS acquisition on the functional profiles obtained using fecal metaproteomics. We identified 24 out of the 26 Clusters of Orthologous Gene (COG) categories for both DDA and DIA datasets with 1,356 and 1,261 COGs, respectively, in this study. As shown in Figure 5, a consistent COG category-level composition of fecal metaproteome was observed regardless of protein digestion method and MS acquisition mode. However, both DDA-PASEF and DIA-PASEF datasets demonstrated that the differential centrifugation dramatically altered the observed functional profiles in metaproteomics. These include the marked decrease in functional category J (translation, ribosomal structure and biogenesis) and N (cell motility) by differential centrifugation, while increased category E (amino acid transport and metabolism), M (cell wall/membrane/envelope biogenesis), R (general function prediction only), and P (inorganic ion transport and metabolism). The decrease in functions related to translation and cell motility suggests that the differential centrifugation may result in an underestimation of microbial species with more active cell proliferation and higher motility. The functional profiles analysis again demonstrated that sample preparation methods need to be optimized for studies with specific objectives or functional pathways of interest.

## DISCUSSION

In this study, our findings showed that DIA acquisition provided a clear advantage compared to DDA for identification and quantification of proteins, including small and antimicrobial proteins/peptides, in microbiome samples. We also demonstrated that non-differential centrifugation methods improved the recovery of small proteins and AMPs, and that FASP workflow using 10kDa molecular cut-off filter achieved similar data outputs compared to in-solution digestion, both of which are commonly used in proteomic and metaproteomic studies. While trying to provide a comprehensive comparison of different experimental steps in metaproteomics, there are still limitations to be considered when continuing this work. Firstly, this study used healthy mouse feces, which might not be representative of human feces, in particular for diseased human fecal samples. To enable the assessment of multiple parameters, we used a pooled mouse fecal sample and technical replicates in this study; the use of biological replicates for further validation is needed and will provide more statistical power. Other sample types can also be tested, such as intestinal content and aspirate samples. Secondly, the current bioinformatic workflow relies on the gene catalog database and DDA data-generated spectral library or reduced protein database, which limits the advantage of DIA-based metaproteomics. This study demonstrated that a library-free search with a full protein sequence database, including those indistinguishable proteins, improved identifications of both gut microbial peptides and taxa. However, it is expected that the use of DDA-independent bioinformatics tools, such as MetaDIA^[43]^, and more comprehensive metagenomic assembled genome (MAG) databases, would benefit the performance of metaproteomics and, thereby, the evaluations of the workflow.

Nevertheless, this study offered a comprehensive comparison of different experimental parameters, including fecal sample pre-processing methods (differential centrifugation and non-differential centrifugation), protein digestion techniques (in-solution and FASP with different molecular cut-off sizes), data acquisition modes (DDA- and DIA-PASEF), and different bioinformatic workflows. We have previously reported that lysis buffer and protein extraction protocols had major impacts on metaproteomics observations, and the protein extraction protocols with strong detergent SDS and ultrasonication achieved the best protein yields and peptide/protein identifications^[32]^. Together with this previous study, we highlighted the critical impact of experimental choices on metaproteomic outcomes and shed light on the potential biases introduced at every step of the workflow. The outcomes of this study provide valuable information in standardizing the metaproteomics workflow for applications, such as clinical study, drug development, and regulatory assessment, especially for microbiome-based medicinal products.

## DECLARATIONS

### Availability of data and materials

All MS proteomics data that support the findings of this study have been deposited to the ProteomeXchange Consortium (http://www.proteomexchange.org) with the data set identifier PXD049086 and PXD049089.

### Copyright

© The Author(s) 2024.
