# Supplementary material for: Assessing fecal metaproteomics workflow and small protein recovery using DDA and DIA PASEF mass spectrometry
Source: Microbiome Res Rep. 2024 Jul 3;3(3):39. doi: 10.20517/mrr.2024.21 (PMC11480776; doi:10.20517/mrr.2024.21)
Supplement: Supplementary file 1 [file mrr-3-3-39-SupplementaryMaterials.pdf]

Supplementary Materials

Assessing fecal metaproteomics workflow and small protein recovery using DDA and DIA PASEF mass spectrometry

Angela Wang[1,2,#], Emily E F Fekete[1,#], Marybeth Creskey[1], Kai Cheng[2,3], Zhibin Ning[2,3], Annabelle Pfeifle[1,2], Xuguang Li[1,2], Daniel Figeys[2,3], Xu Zhang[1,3]

[1]Regulatory Research Division, Biologic and Radiopharmaceutical Drugs Directorate, Health Products and Food Branch, Health Canada, Ottawa K1A 0K9, Ontario, Canada.

[2]Department of Biochemistry, Microbiology and Immunology, Faculty of Medicine, University of Ottawa, Ottawa K1H 8M5, Ontario, Canada.

[3]School of Pharmaceutical Sciences, Faculty of Medicine, University of Ottawa, Ottawa K1H 8M5, Ontario, Canada.

[#]Authors contributed equally.

Correspondence to: Dr. Xu Zhang, Regulatory Research Division, Biologic and Radiopharmaceutical Drugs Directorate, Health Products and Food Branch, Health Canada, 251 Sir Frederick Banting Driveway, Ottawa K1A 0K9, Ontario, Canada. E-mail: xu.zhang@hc-sc.gc.ca

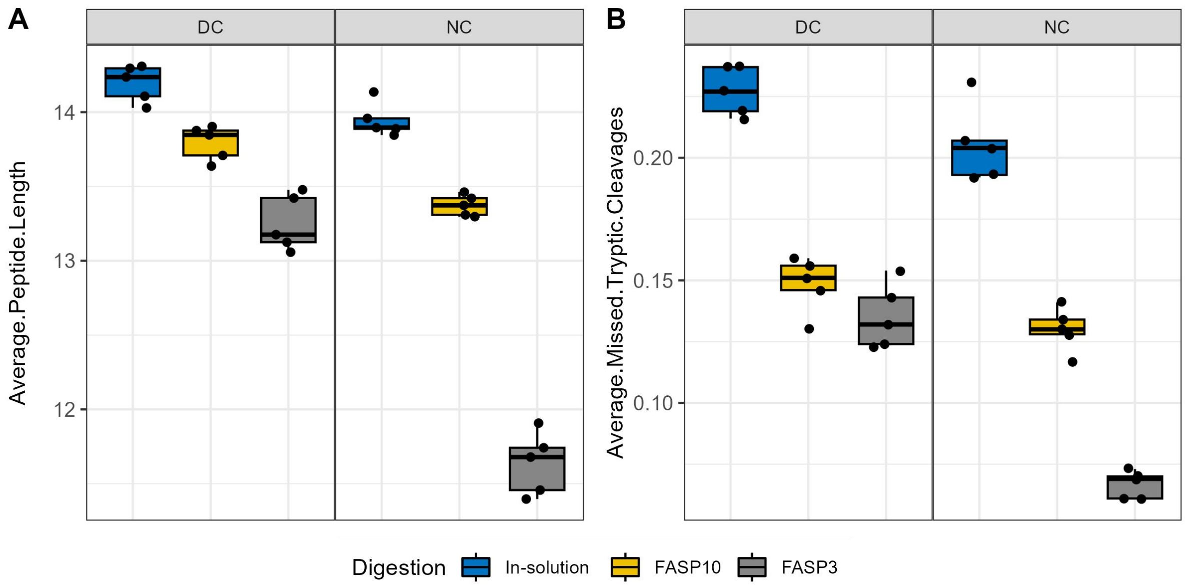

Supplementary Figure 1. Boxplot demonstrating the average length (A) and number of missed tryptic cleavages (B) of identified peptides in different groups of DIA-PASEF data.

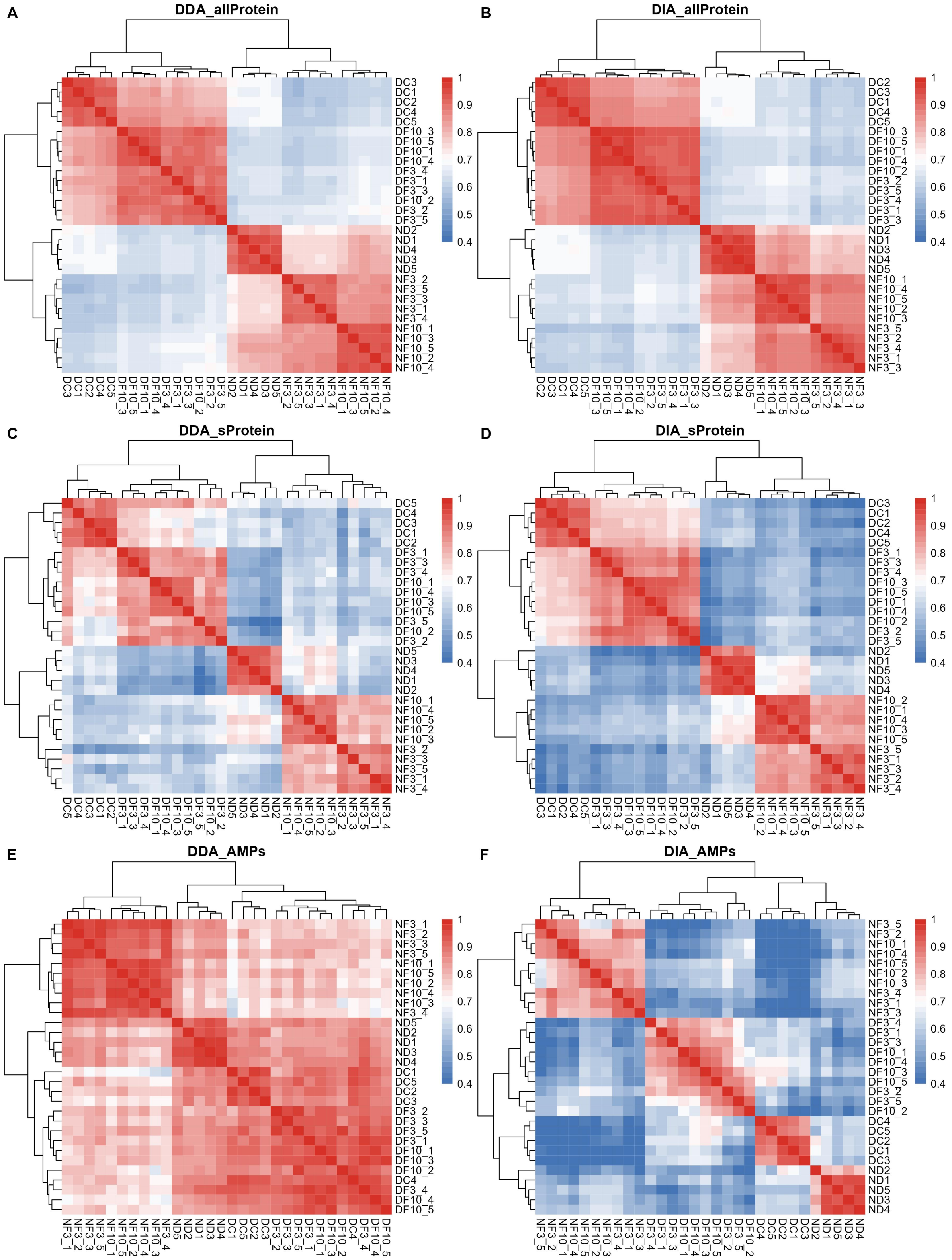

Supplementary Figure 2. Heatmap of Pearson's correlation r between samples. Pearson's correlation r was calculated sample-wise with quantified intensities (log-10 transformed) of all proteins (A and B), small protein only (C and D), and identified AMPs (E and F). Pearson's correlation was calculated with R function *cor* (use = "pairewise.complete.obs") and plotted with *pheatmap*.

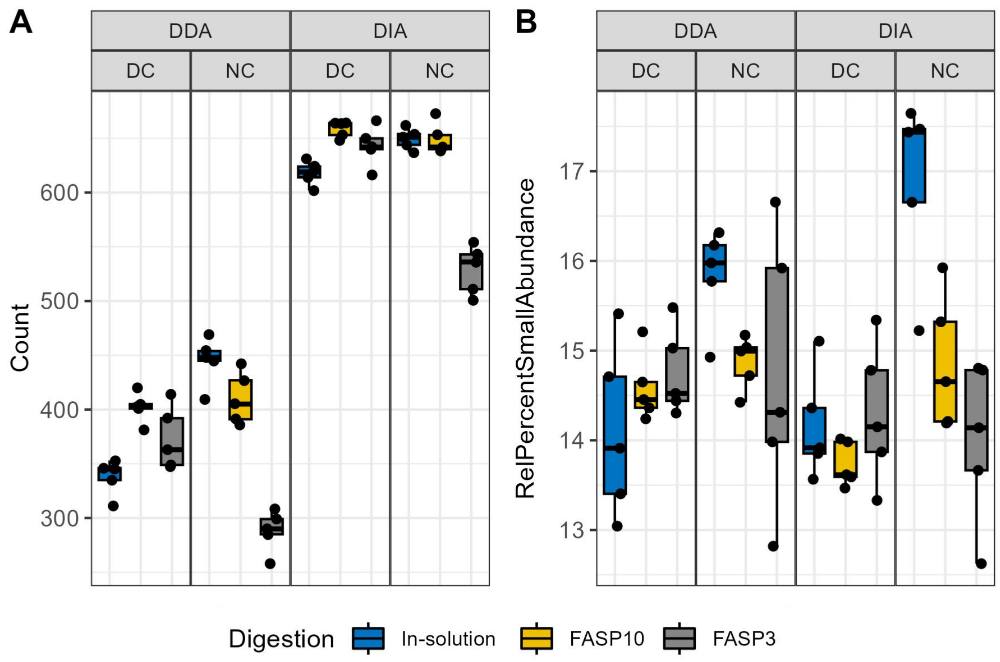

Supplementary Figure 3. Identification and abundance of host proteins in mouse fecal samples. Boxplots showing the number (A) of host proteins identified and their relative abundance (B) within the sample.

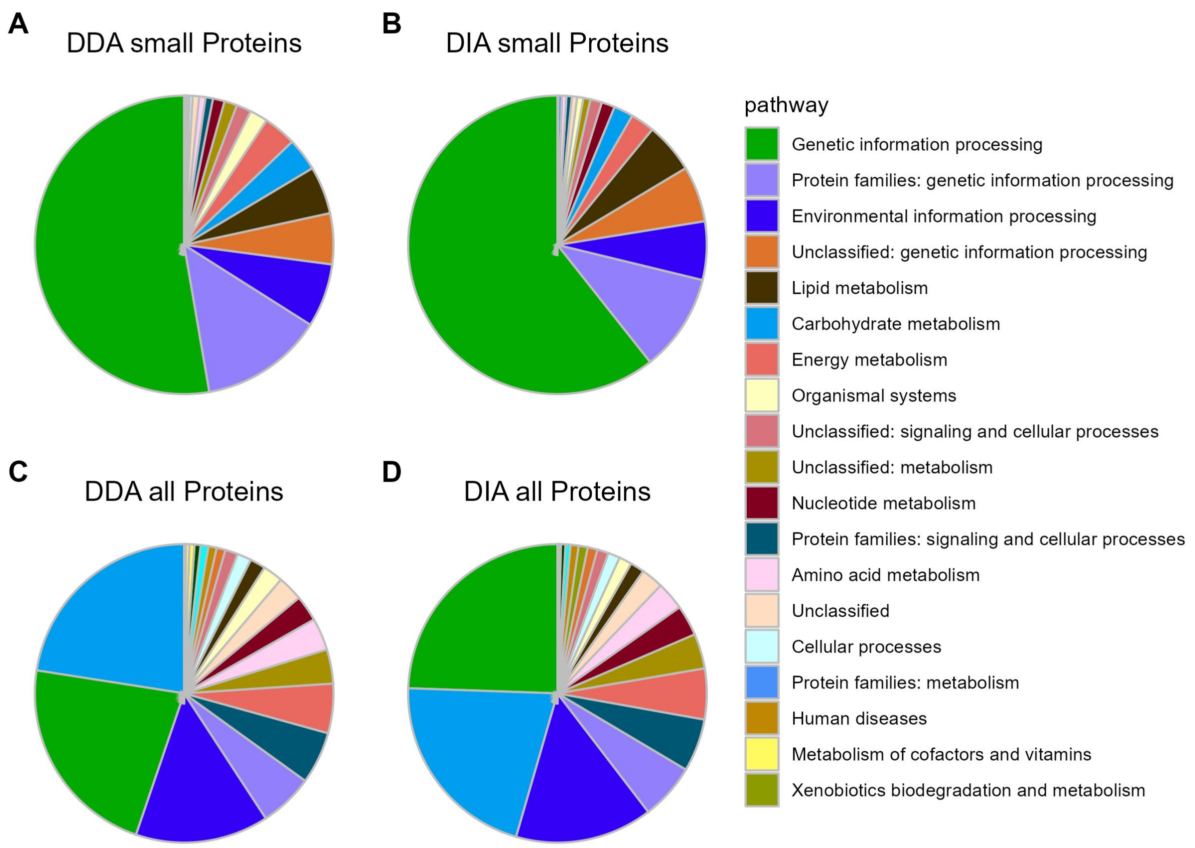

Supplementary Figure 4. GhostKOALA functional annotation of small proteins identified (≤ 100 amino acids) (A and B) as well as all proteins identified (C and D) in DDA and DIA datasets.

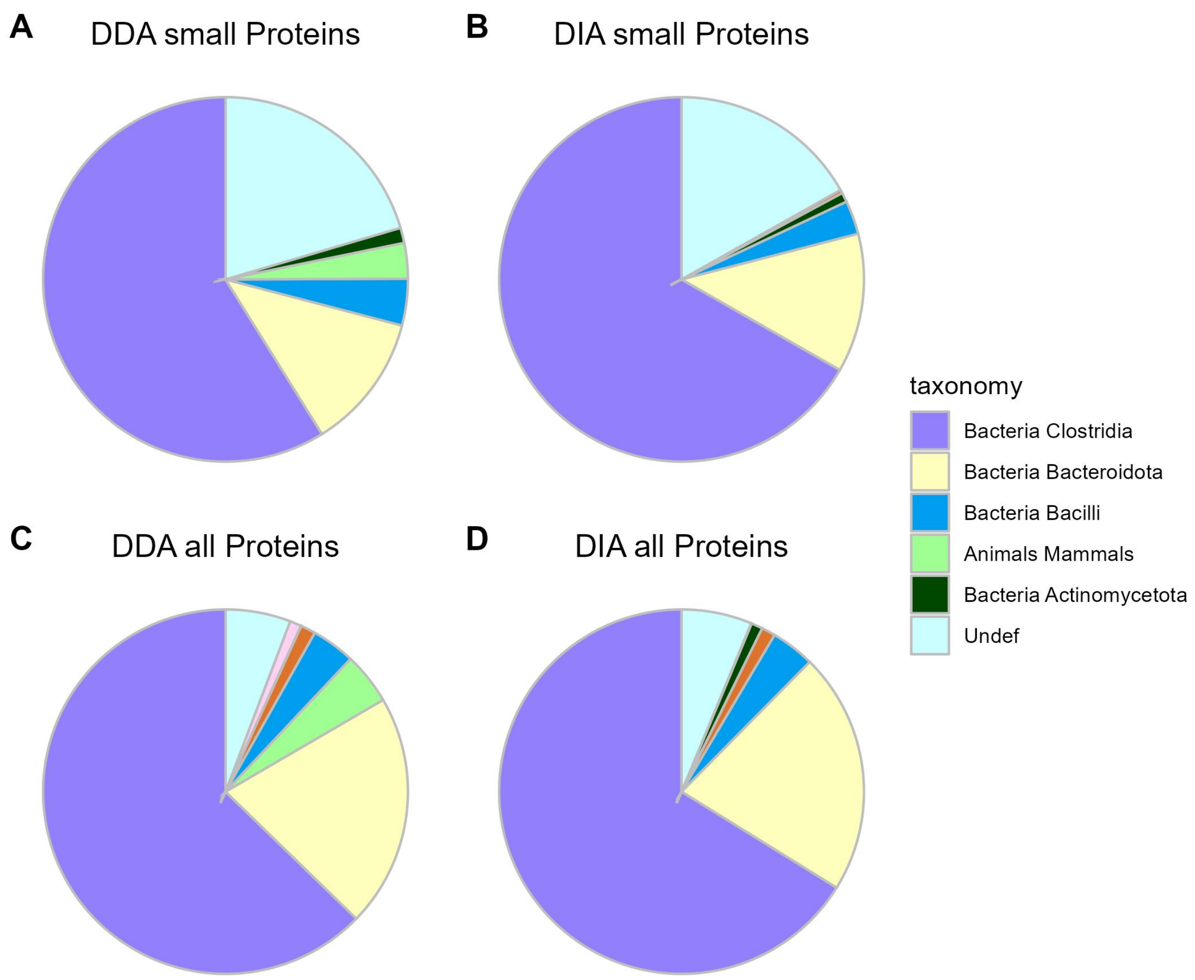

Supplementary Figure 5. GhostKOALA taxonomic annotation of small proteins identified (≤ 100 amino acids) (A and B) as well as all proteins identified (C and D) in DDA and DIA datasets.

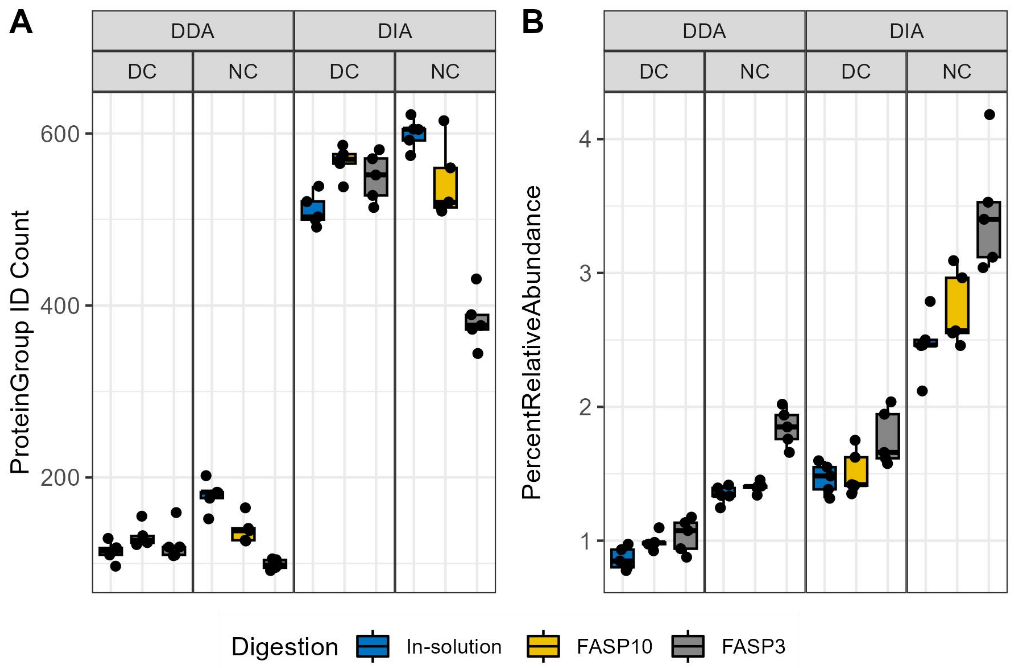

Supplementary Figure 6. Identification of small proteins in samples when searched against a small protein database. Count of identified small proteins (A). Percent relative abundance of small proteins compared to total abundance of proteins in sample (B).

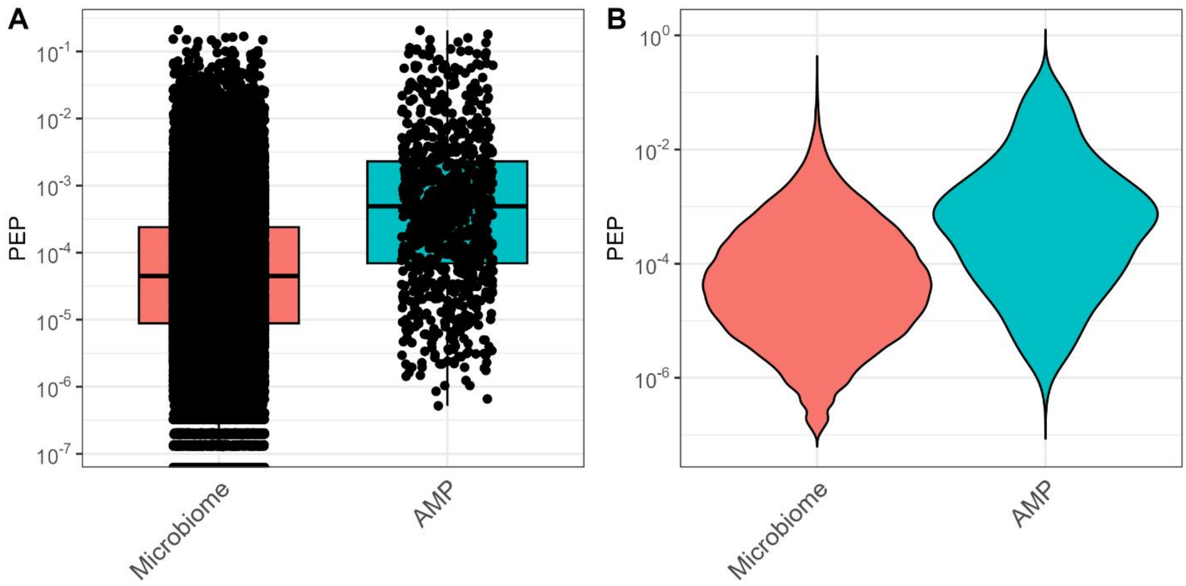

Supplementary Figure 7. Boxplot showing the PEP (posterior error probability) scores of microbial or AMP peptides (A) and violin plot of PEP values to show PEP score distribution of microbial or AMP peptides (B).

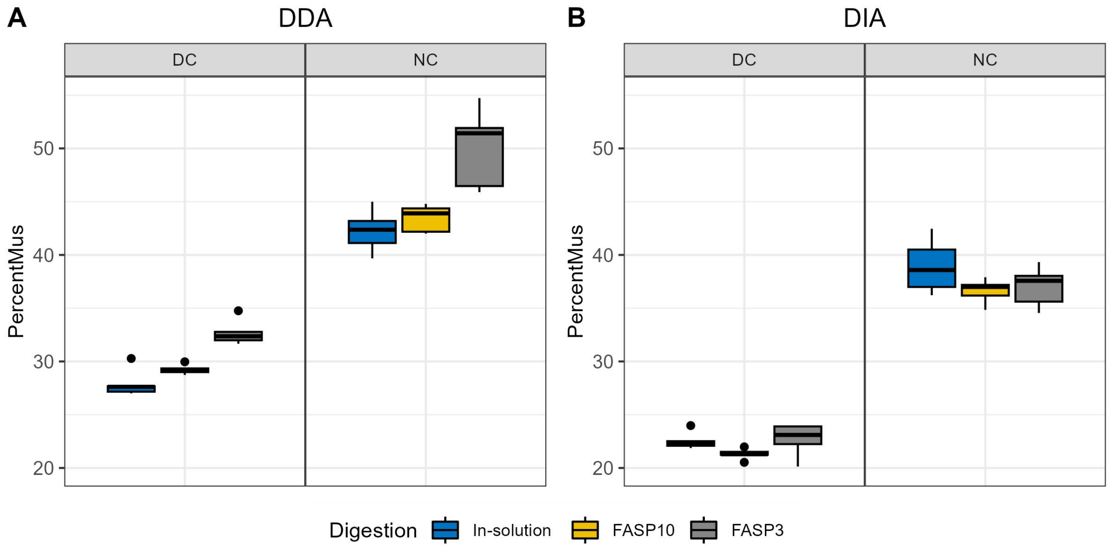

Supplementary Figure 8. Boxplot showing the relative abundance of the genus Mus (host) quantified in each group for DDA (A) and DIA (B) data during taxonomic analysis. Two-way ANOVA was performed for each dataset with differential centrifugation and digestion method as the two factors. In DDA data (A), significant differences were observed for both differential centrifugation (*P* value $2x10^{-16}$) and digestion method (*P* value $4.8x10^{-7}$). In DIA data (B), significant difference was observed for differential centrifugation (*P* value $2x10^{-16}$) but not for digestion method (*P* value 0.0605).
